# Polarization-directed growth of spiral nanostructures by laser direct writing with vector beams

**DOI:** 10.1038/s41467-023-37048-0

**Published:** 2023-03-14

**Authors:** Xiaolin Lu, Xujie Wang, Shuangshuang Wang, Tao Ding

**Affiliations:** Key Laboratory of Artificial Micro/Nano Structure of Ministry of Education, School of Physics and Technology, Wuhan University, Wuhan, 430072 China

**Keywords:** Laser material processing, Nanophotonics and plasmonics, Nanophotonics and plasmonics

## Abstract

Chirality is pivotal in nature which attracts wide research interests from all disciplines and creating chiral matter is one of the central themes for chemists and material scientists. Despite of significant efforts, a simple, cost-effective and general method that can produce different kinds of chiral metamaterials with high regularity and tailorability is still demanding but greatly missing. Here, we introduce polarization-directed growth of spiral nanostructures via vector beams, which is simple, tailorable and generally applicable to both plasmonic and dielectric materials. The self-aligned near field enhances the photochemical growth along the polarization, which is crucial for the oriented growth. The obtained plasmonic chiral nanostructures present prominent optical activity with a g-factor up to 0.4, which can be tuned by adjusting the spirality of the vector beams. These spiral plasmonic nanostructures can be used for the sensing of different chiral enantiomers. The dielectric chiral metasurfaces can also be formed in arrays of sub-mm scale, which exhibit a g-factor over 0.1. However, photoluminescence of chiral cadmium sulfide presents a very weak luminescence g-factor with the excitation of linearly polarized light. A number of applications can be envisioned with these chiral nanostructures such as chiral sensing, chiral separation and chiral information storage.

## Abstract (web summary)

Chiral nanostructures are in demand for various applications, but facile and scalable fabrication is a technical challenge. Here, the authors report polarization-directed chiral growth of complex spiral patterns by laser direct writing with vector beams.

## Introduction

Chiral nanostructures have played an important role for enhanced chiral light–matter interactions, which is particularly crucial in chiral molecule sensing^1^, enantiomer separation^2^, disease diagnosis and treatment^3,4^, and chiral light manipulation^5^. Although tremendous efforts have been devoted to the design and fabrication of chiral nanostructures^6–8^, state-of-the-arts strategies possess drawbacks that cannot meet all the demands on simplicity, regularity, tailorability, scalability, and generality, which are essential for the translation^9–11^. Although chiral nanomaterials synthesized via wet chemistry or self-assembly are scalable and tuneable, their sensing capability is limited to a specific chiral substance^12,13^. Fabrication via physical methods such as nanolithography (including E-beam, imprint and colloidal lithography), glancing angle deposition (GLAD) can achieve clean chiral metasurfaces with moderate uniformity and regularity^14^, however, the high cost of the instrumentation and the complication of the operation make them hard for commercialization^15^.

Alternatively, fabrication of chiral nanostructures via laser direct writing is much facile but largely restricted to polymer based materials^16,17^. Thus, additional procedure such as metal doping, evaporation, or electroplating^18–20^ has to be employed to improve the chiral light–matter interactions, which incurs the complication of the fabrication. Although helical metallic nanostructures can be fabricated via vortex beam^21–23^, they are mostly in the range of microns due to the wavefront modulation in three dimensional (3D) space, which shifts the chiroptic response in the infrared. Refined chiral nanostructures with strong light–mater interaction in the visible region can hardly be achieved via conventional optical approach. Moreover, most of the strategies mentioned above are only suitable for chiral structures made of one type of materials, which significantly compromises their generality.

Polarization-directed growth offers exceptional control on material orientation in subwavelength scale^24–26^. Although chiral structures addressed with circular polarized light is potentially possible in theory^27,28^, additional support of the chiral near field by nonspherical nanoparticles (NPs) is required^29^.

**Fig. 1:**
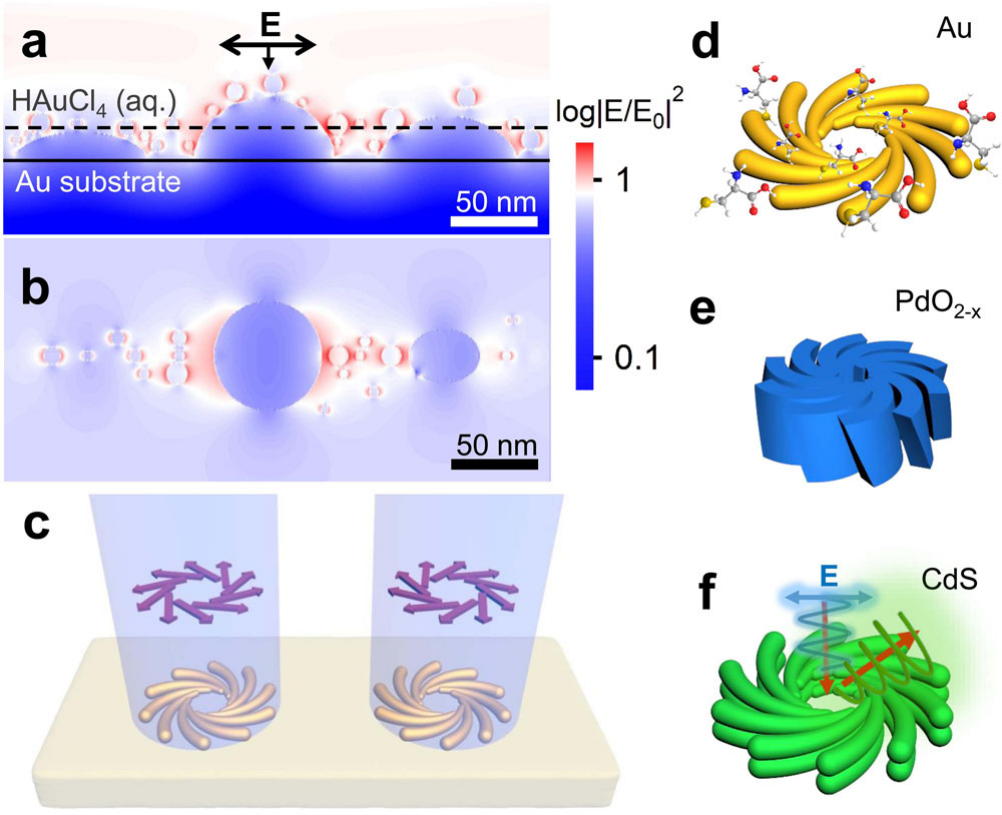
Concept of polarization-directed growth of chiral nanosturtures. **a**, **b** Near field profiles of Au NPs (diameter: 8 ± 4 nm) on Au films. **a** Cross-section view, **b** Top view. The big ribbons are roughened Au films induced by etching and the small spheres are Au nanoseeds generated by the laser irradiation. The solid line represents the surface of Au films and the dashed line indicates the cross-section of the top view shown in (**b**). **c** Schematic of spiral nanostructures formed by laser direct writing with vector beams. The purple arrows represent the polarization directions of the vector beam (light blue). **d**–**f** Schematic of spiral nanostructures: **d** Au for chiral molecule sensing, **e** PdO_2−*x*_ for dielectric metasurfaces, and **f** CdS for chiral photoluminescence.

Here, we introduce a polarization-directed chiral growth of inorganic nanomaterials, which is seedless and ligand-free, making them ideal sensing platform of chiral enantiomers. The inorganic precursor solution undergoes photochemical redox reactions under continuous wave (CW) laser irradiation, which generates nanoseeds and roughened substrate at the focal spot. These tiny seeds and the roughened substrate provide the self-aligned near fields for boosted growth of the nanoparticles along the polarization (Fig. 1a, b). By engineering the polarization profile with vector beams, the oriented growth can evolve into chiral configuration with controlled spirality (Fig. 1c), which is applicable to both planar plasmonic and quasi-3D dielectric materials (Fig. 1d–f). This tailorable fabrication process can be easily implemented based on conventional laser direct writing setup with affordable scalability and excellent regularity, which offers a plethora of opportunities for chiral sensing, separation, and information storage.

## Results

### Polarization-directed oriented growth of Au nanospindles

**Fig. 2:**
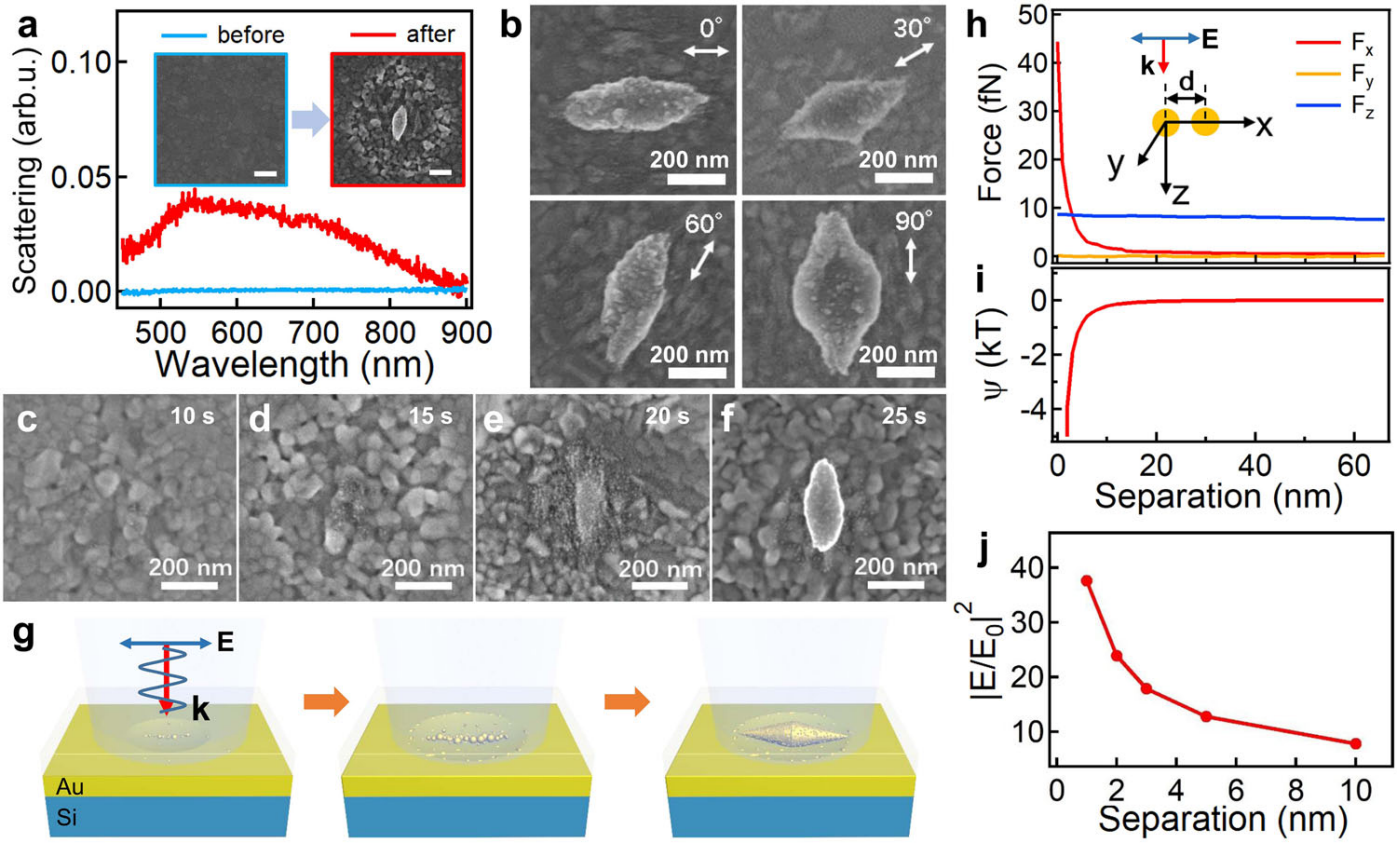
Polarization-directed oriented growth of Au nanospindles. **a** Change of DF scattering spectra before and after irradiation with 446 nm CW laser (4 mW, 15 s). Insets are the corresponding SEM images, scale bars are 200 nm. **b** SEM images of Au nanospindles with orientation changing with the direction of polarization. **c**–**f** Evolution of Au nanospindles with time of irradiation. **g** Schematic of the polarization-directed growth process. **h** Optical force (*F*_opt_) and **i** energy (Ψ) analysis of Au NPs with different separation. **j** Change of near field intensity with the separation of the Au NPs (diameter: 14 nm, the typical size of Au NPs found in SEM images). Source data are provided as a Source Data file.

Precursors of noble metals such as HAuCl_4_, AgNO_3_, K_2_PdCl_6_, etc. can be photochemically reduced under intense laser irradiation, which normally form insoluble nanoparticles in solution (see Supplementary Note 1)^30,31^. Here we perform the irradiation of HAuCl_4_ solution on Au films under optical dark field (DF) microscope and strong plasmonic scattering emerges after the irradiation, suggesting the growth of nanospindles as confirmed by the scanning electron microscope (SEM) images (Fig. 2a and insets). The orientation of the nanospindles can be easily adjusted by changing the laser polarization angle with high fidelity (Fig. 2b and Supplementary Fig. 1a, b). The Au spindles show polarization-dependent plasmonic response of the longitudinal mode while the transverse mode is less sensitive to the polarization likely due to the roughened Au surfaces (Supplementary Fig. 1c). In contrast, only spherical aggregates of Au NPs are generated with the irradiation of circularly polarized light, which shows no polarization-dependent scattering (Supplementary Fig. 1d). It is noted that the surface around the spindle is roughened after the irradiation (inset of Fig. 2a) and the snapshots of the growth process reveal the Au films are etched at initial stage of the irradiation, followed by producing tiny Au nanoseeds in the beam spot (Fig. 2c, d). The laser polarization seems to connect the Au nanoseeds with oriented growth, which eventually form the nanospindles (Fig. 2e, f). Higher irradiation power however results in bulk growth of large Au crystal over the spindle (Supplementary Fig. 2). The whole growth process is schematically illustrated in Fig. 2g. The optical forces (see Fig. 2h and Supplementary Note 2) here may take a minor role as the optical dipole interaction (≈40 fN, Fig. 2h) is smaller than the Brownian forces (≈pN)^32^. The Au nanoseeds are prone to aggregate due to the strong van de Waals interaction when the separation is smaller than 2 nm (Fig. 2i and Supplementary Note 3). Clearly, such a destabilization is promoted by the fast growth kinetics along the polarization as the electric field increases exponentially with the decrease of gap size (Fig. 2j)^33^. This polarization-directed anisotropic growth can be rationalized that HAuCl_4_ oxidizes the Au films with laser irradiation^34^, which results in increased surface roughness (Supplementary Fig. 3). Such roughened Au substrate increases the number of local hot spots (Fig. 1a, b and Supplementary Fig. 4), which promotes the plasmon-enhanced photoreduction of HAuCl_4_ along the polarization^24^, leading to the oriented growth of nanospindles. The temperature increase of the Au films during the irradiation is less than 10 °C, which has a negligible effect on the growth kinetics (Supplementary Fig. 5). Thus, the concentrated near fields supported by the surface plasmons are crucial for such a polarization-directed anisotropic growth. In contrast, for flat substrate such as Si, only spherical Au NPs were generated after the irradiation (Supplementary Fig. 6). Another requirement for such polarization-directed growth is the precursor needs to be photochemically active (Supplementary Fig. 7). Similarly, we observe several other inorganic materials can also anisotropically grow along the direction of laser polarization, such as PdO_2−*x*_ and CdS (see Supplementary Note 1 for the detailed discussion). Indeed, their precursors are photochemically reactive^35^, and the snapshots of their growth process all show that the oriented growth emerges based on the granulated NPs films formed at early stage of the growth (Supplementary Figs. 8, 9). However, the near field enhancement of these dielectrics is not as strong as that of Au, thus, other forces such as optical dipole interactions may be influential in this particular case^36^.

### Polarization-directed growth of spiral nanostructures via vector beams

**Fig. 3:**
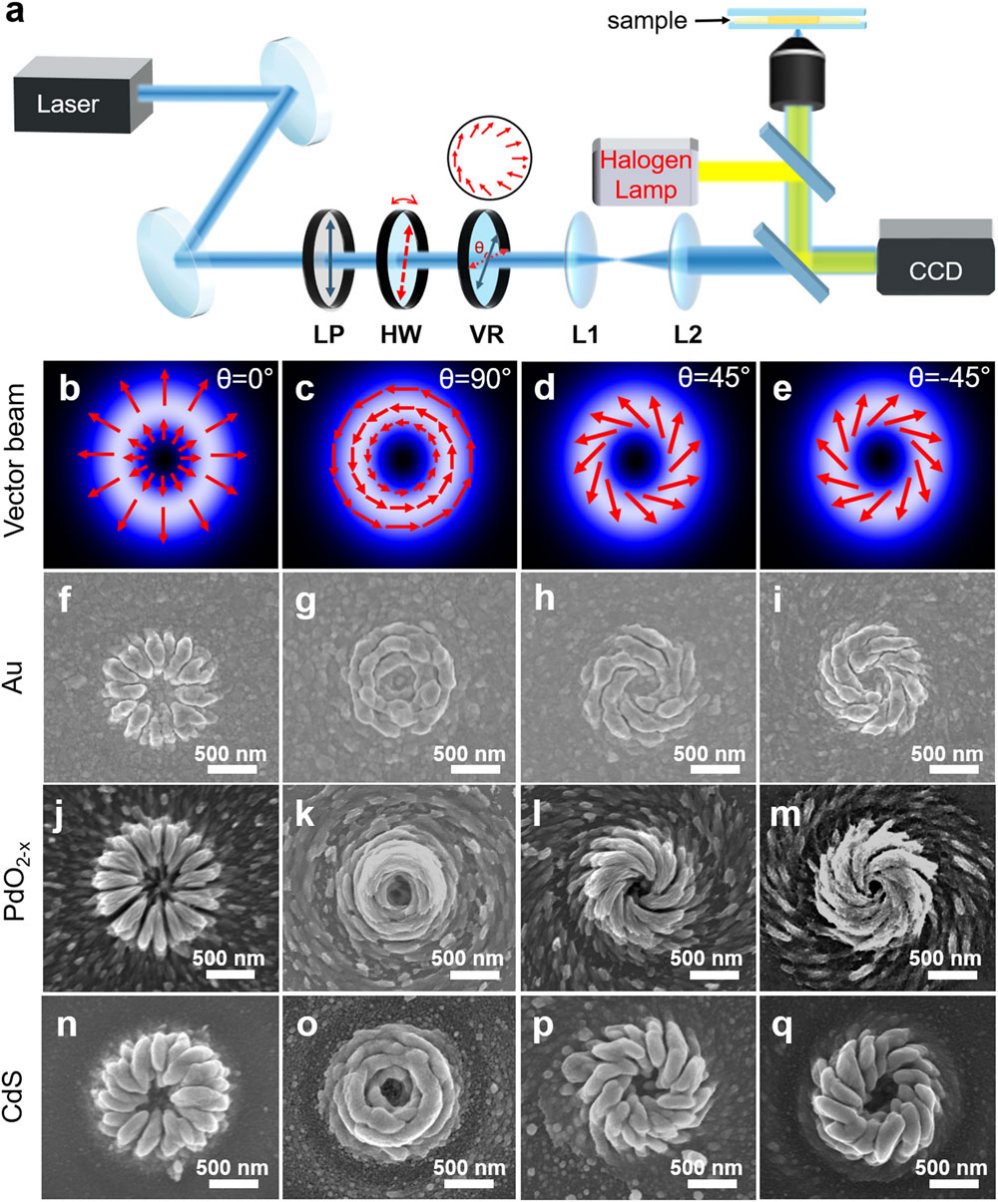
Polarization-directed growth of complex (chiral) patterns via vector beams. **a** Schematic of experimental setup. LP: linear polarizer. HW: half-wave plate. VR: vortex retarder, which can create polarization vortices. The red arrows in the circle represent the distribution of polarization direction on the vortex plate with red dots indicating the fast axis. Blue line represents the polarization and red line represents the fast axis of the VR. L1/L2: beam expander. **b**–**e** Simulated beam profile with different vectors. The angle (θ) between the polarization and the fast axis of VR are **b** 0°, **c** 90°, **d** 45°, **e** −45°. The red arrows are indicators for polarization orientation. **f**–**q** Corresponding SEM images of the complex (chiral) patterns formed with different vector beams. **f**–**i** Au, **j**–**m** PdO_2−*x*_, **n**–**q** CdS.

Since the growth is directed by laser polarization we can potentially obtain arbitrary shapes as long as the polarization direction is engineered properly. Here, we use tailored vector beam^37,38^ via aligning the polarization and the fast axis of vortex retarder (VR) in different angles (θ) to obtain different polarization patterns (see Fig. 3a–e and Supplementary Note 4 for detailed discussion) so that the growth follows the exact polarization patterns, which generates complex nanostructures of different inorganic materials (Fig. 3f–q and Supplementary Fig. 10). The longitudinal component of the focused vector beam^39,40^ clearly does not take any obvious effect on the growth as the generated nanostructures all appear hollow in the center. Particularly, the focused spiral beam also known as Kevin’s chirality^41^ can create chiral dipole moment inside the medium^42^, which guides the oriented growth of spiral nanostructures. The diameter of the spiral nanostructures can be tuned with different magnification objectives (Supplementary Fig. 11) and the smallest spacing between individual nanostructures needs to be larger than 2.5 μm to avoid interference during the laser-induced growth. The number of needles appeared within the nanostructure is different for different materials and polarizations. Faster growth kinetics (like Au) can result in lateral growth and subsequently merging of the chiral arms, which leads to decreased number of needles in the nanostructures. The chiral nanostructures fabricated in this way show prominent advantages of generality, tuneability, simplicity, regularity, and structural uniformity compared to other methods^43,44^, which can potentially be applied for chiral molecules sensing, chiral metasurfaces, chiroptics, and chiral information storage.

### Chiral sensing based on chiral plasmonic nanostructures

**Fig. 4:**
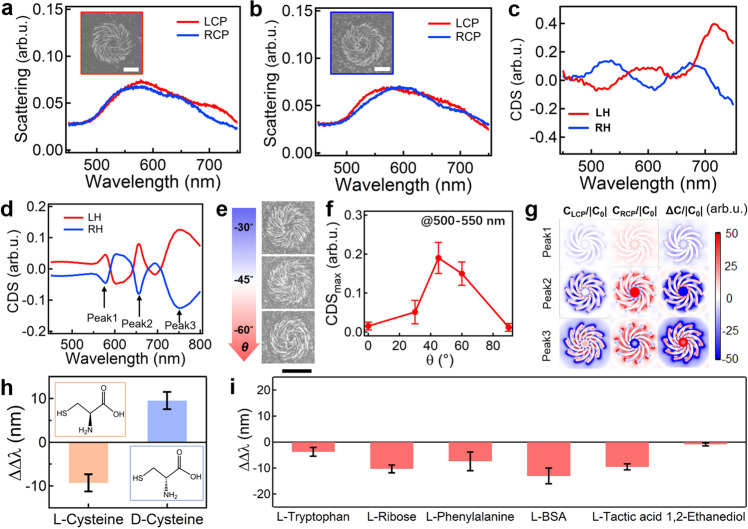
Chiroptic property of chiral Au nanostructures and their applications for chiral molecule sensing. Scattering spectra of **a** LH and **b** RH chiral nanostructures with LCP and RCP incidences, and their corresponding **c** CDS spectra. Insets are the corresponding SEM image of the LH and RH enantiomers. Scale bars are 500 nm. **d** Simulated CDS spectra of LH and RH chiral nanostructures. **e** SEM images of chiral nanostructures with increasing θ. Scale bar is 1 μm. **f** Change of CDS intensity (peaks@500-550 nm) with θ. **g** Near field optical chirality of different modes marked in (**d**). Sensing application of the chiral plasmonic enantiomers: **h** Spectral dissymmetric factors of RH and LH induced by L- and D-cysteine. Insets are their chemical structures. The orange and blue colors denote the enantiomers of L- and D-cysteine, respectively. **i** Spectral dissymmetric factors of RH and LH induced by L-Tryptohan, L-Ribose, L-Phenylalanine, L-BSA, L-Tactic acid, and 1,2-Ethanediol, respectively. All the error bars shown in this figure are standard deviation based on the measurements of 3 sets of samples. Source data are provided as a Source Data file.

The unique advantages of this polarization-directed chiral growth are that not only the structural chirality can be easily tuned via changing the alignment angle (θ) but also their surfaces are ligand-free, which is crucial for sensing of different chiral analytes via superchiral fields^45^. Here the Au chiral nanostructures with anti-clockwise (left-handed, LH) and clockwise (right-handed, RH) rotation show different response to left-/right-circularly polarized (LCP/RCP) light (Fig. 4a, b), which leads to opposite response of circular differential scattering (CDS, Fig. 4c). These Au chiral nanostructures show some variability in the CDS spectra for different individuals (Supplementary Fig. 12) which is commonly observed due to the minute structural differences incurred by the fabrication tolerance^46^. Simulation of similar chiral plasmonic nanostructures (Supplementary Fig. 13) shows multiple modes (peak 1–3) in the range from 500 to 800 nm (Fig. 4d), which agrees with the experimental results (Fig. 4c). The deviation in experimental spectra is mainly due to the imperfection of surface roughness and geometry of the patterns. The chirality of the plasmonic spiral structures can be tuned by changing the alignment angle (θ, Fig. 4e), which shows the maximum CDS intensity at the angle of 45° (Fig. 4f and Supplementary Fig. 14).

The optical chirality (*C*) of the plasmonic nanostructures is defined in Eq. (1)

1 $$\complement=\frac{1}{2}\left[{\varepsilon }_{0}{{{{{\bf{E}}}}}}\cdot \left(\nabla \times {{{{{\bf{E}}}}}}\right)+\frac{1}{{\mu }_{0}}{{{{{\bf{B}}}}}}\cdot \left(\nabla \times {{{{{\bf{B}}}}}}\right)\right]$$

where **E** and **B** are the electric and magnetic fields, respectively. Here we define $\triangle C/{{|C}}_{0} |=({C}_{{{{{{\rm{LCP}}}}}}}-{C}_{{{{{{\rm{RCP}}}}}}})/|{C}_{0}|$, where *C*_0_ is field of chiral incidences without chiral structures^47^. Clearly, such a spiral nanostructure presents prominent superchiral fields with large enhancement over 50 (Fig. 4g), which predicts large dissymmetric factor for chiral sensing applications^34^.

Here we adopt these chiral enantiomers of LH and RH plasmonic nanostructures for chiral analytes sensing based on their induced spectral dissymmetry of CDS. The adsorption of the chiral molecules on the chiral plasmonic nanostructures results in different magnitudes of the plasmon resonance shift (∆*λ*_LH_ and ∆*λ*_RH_) due to asymmetric modification of local refractive index^45^. Since the chirality can be characterized by the g-factor which is also defined as 2ΔΔλ/(Δλ_LH_ + Δλ_RH_), this dissymmetric factor can be parameterized using $\triangle \triangle {{{{{\rm{\lambda }}}}}}=\triangle {\lambda }_{{{{{{\rm{LH}}}}}}}-\triangle {\lambda }_{{{{{{\rm{RH}}}}}}}$^48–51^, which shows magnitude up to ≈9 ± 1.95 nm for cysteine (Fig. 4h and Supplementary Fig. 15). The detection concentration of the chiral molecules (L-cysteine) can be as low as 0.01 mg mL^−1^ whose dissymmetric factor is reduced to ≈2 ± 1.5 nm (Supplementary Fig. 16). This chiral plasmonic chip can be reused for the analysis of other chiral molecules as long as it is not polluted (Fig. 4i and Supplementary Fig. 17). Clearly, the L-enantiomers all show negative dissymmetric factors which can be used to discriminate the chirality of the molecules. In contrast, for 1,2-Ethanediol with no chirality, the dissymmetric factor appears zero, which indeed suggests the validity of the measurement.

### Chiral metasurfaces based on dielectric materials

**Fig. 5:**
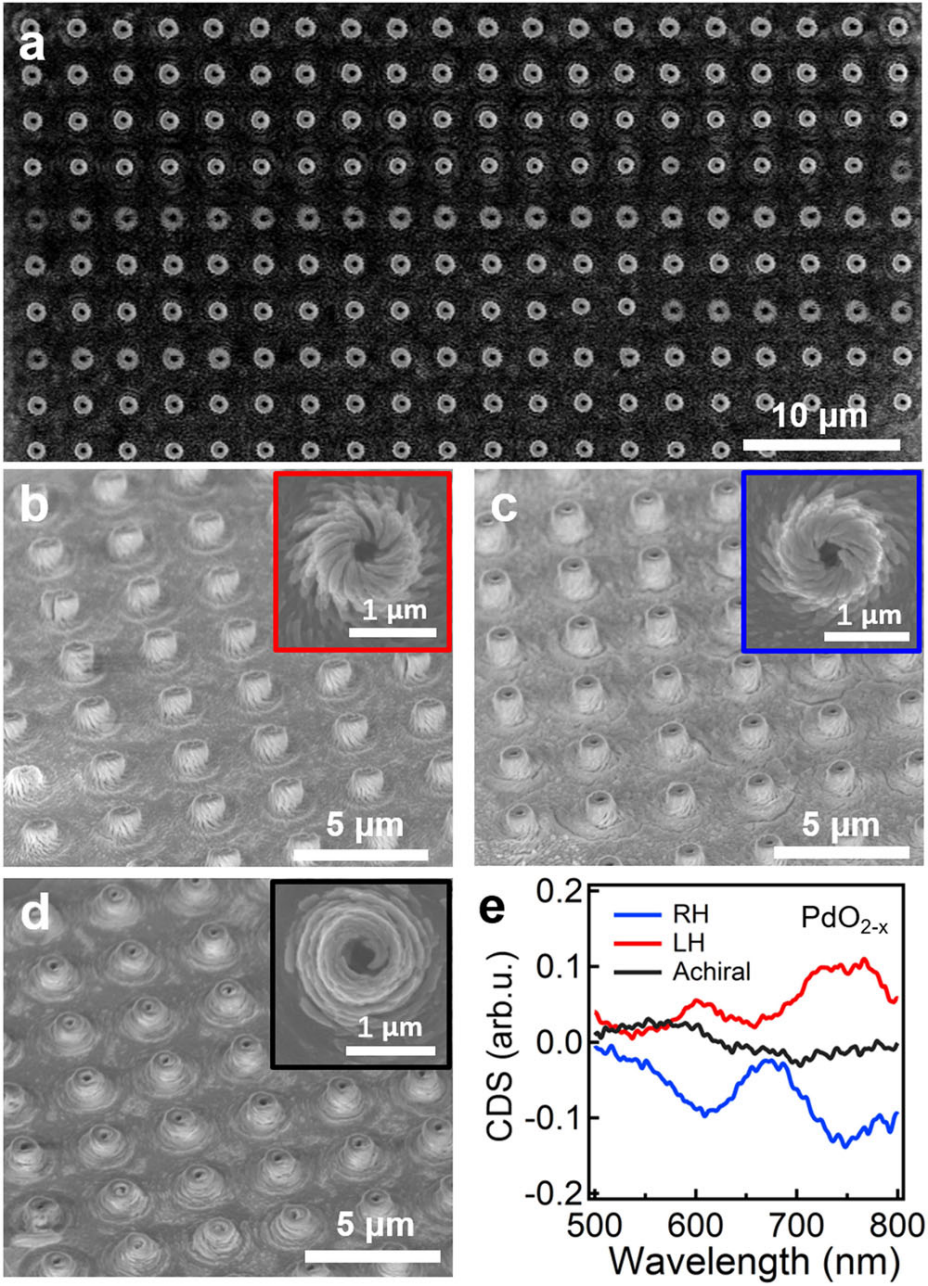
Chiroptic property of chiral PdO_2−__x_ nanostructures. **a** SEM image of RH PdO_2−x_ arrays in sub-millimeter scale. **b**–**d** SEM images of PdO_2−__x_ nanoarrays with **b** LH, **c** RH, and **d** achiral configurations. **e** CDS spectra of the PdO_2−__x_ nanoarrays shown in **b**–**d**. Source data are provided as a Source Data file.

Another advantage of this polarization-directed growth is that it can be generally applicable for the growth of other kinds of inorganic materials such metal oxides and metal sulfide, which are exploitable for chiral dielectric metasurfaces and luminescence devices. Unlike ebeam lithography, the chiral metasurfaces can be easily fabricated via laser direct writing in sub-mm scale (Fig. 5a), which is simple and cost-effective. Both LH and RH PdO_2−X_ chiral nanostructures present quasi-3D chiral configuration (Fig. 5b, c) with a g-factor reaching up to 0.1 in the visible region (Fig. 5e) while for achiral metasurfaces (Fig. 5d), the g-factor is smaller than 0.02 due to the chromatic aberration of polarization optics (Supplementary Fig. 18).

**Fig. 6:**
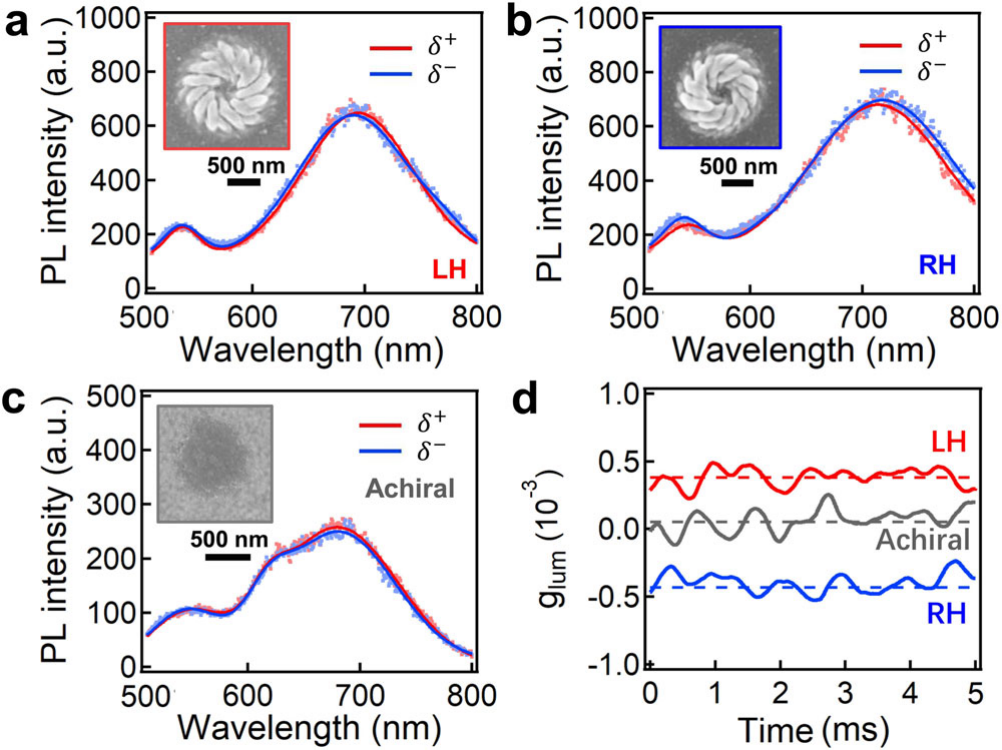
Chiral PL property of chiral CdS nanostructures. Chiral PL spectra of CdS nanostructures with **a** LH, **b** RH and **c** achiral configurations. Dots are the raw data and solid lines are fitted data. Light (473 nm) of LP was applied as the excitation source. *δ*^+^ and *δ*^−^ represent the detected signals with LCP and RCP waveplates, respectively. SEM insets represent the typical samples of enantiomers and achiral ones. **d** Chiral PL *g*-factor (*g*_lum_) of RH, LH and achiral nanostructures. Note the *g*_lum_ of achiral CdS is normalized to zero. Source data are provided as a Source Data file.

For metal sulfide with direct band gap, such as CdS, their photoluminescence (PL) can potentially present chiral emission due to its interaction with the chiral nanostructures. However, we observe very tiny difference in the chiral PL with the excitation of linearly polarized (LP) light, which is largely because no amplification mechanism (such as Mie resonance) exists in such chiral nanostructures (Fig. 6a, b). As for achiral samples, their chiral PL appears almost identical (Fig. 6c). To improve the detection sensitivity, we employ photomultiplier tube to measure the PL intensity in the range from 600 to 700 nm, and the results indeed show a very small difference in the optoelectronic signals when detecting with LCP and RCP waveplates (Supplementary Fig. 19), which gives a very small luminescence g-factor (*g*_lum_) of ≈4.0 × 10^−4^ (Fig. 6d). Although chiral excitation can potentially steer much larger *g*_lum_^36, 52^, an enhancing mechanism of such chiral emission is desired for practical applications, which remains much of future efforts.

## Discussion

In summary, we introduced a polarization-directed chiral growth of different inorganic materials by utilizing the vector beam. Such a polarization-directed growth is established on the pronounced near field enhancement of the roughened substrate and proper photochemistry of the inorganic precursors. The clean surfaces of the chiral nanostructures facilitate the detection of chiral substances with relatively large spectral dissymmetric factor of ≈10 nm. For chiral semiconductors such as CdS, it only generates very weak chiral light emission with LP excitation, which requires further improvement. Nevertheless, it provides a paradigm of polarization-directed chiral nanostructures, which significantly expands the family of chiral nanomaterial and fabrication methods. Besides the chiral sensing capability shown in this work, it can be envisaged that a wide range of applications based on these chiral nanostructures will be exploited such as chiral metasurfaces, chiral separation, chiral catalysis and information storage based on optical chirality^53–56^.

## Methods

### Materials

Gold chloride hydrate (HAuCl_4_·4H_2_O, 99.95%), potassium hexachloropalladate (K_2_PdCl_6_, 99%), L-Phenylalanine (99%) were purchased from Sigma-Aldrich. L-Cysteine (99%), L-Ribose (99%) and Bovine Serum Albumin (BSA, 96%) were obtained from Macklin, Accela and bidepharm, respectively. All other chemicals (99%) were provided by Sinopharm unless stated. Deionized (DI) water (from Millipore) was used for all the solution preparation. Au and Pt films with thickness of 70 nm were fabricated on Si substrate via thermal evaporation (JSD-400) or DC magnetron sputtering (Nexdep, Angstrom Engineering Inc.).

### Polarization-directed oriented growth of inorganic NPs

A LP CW laser of 446 nm was focused on the samples through a 100× DF objective (Olympus BX35, NA = 0.8). The samples made of Au/Si (Pt/Si) or bare Si substrates were immersed in precursor solutions of certain concentrations with coverslip for the laser irradiation. Specifically, to obtain Au spindles, Au substrates were immersed in 10 mM HAuCl_4_ solution and irradiated at 4 mW for 20 s. The polarization direction of the laser can be changed by rotating a half-waveplate in the beam path. To obtain PdO_2−x_ ellipsoids, Pt substrates were immersed in K_2_PdCl_6_ solution (5 mM) and irradiated at 0.5 mW for 10 s. For CdS ellipsoids, laser irradiation was performed on Si substrates immersed in the solution of CdCl_2_ (2 mM)/Na_2_S_2_O_3_ (100 mM). The irradiation power is ≈2 mW with duration of ≈10 s. After the irradiation, all the samples were rinsed with DI water and blow-dried with nitrogen gun for further characterizations.

### Polarization-directed chiral growth of inorganic nanostructures

The polarization of the CW laser was attenuated to be radially and azimuthally polarized by aligning the polarization along and perpendicular to the fast axis of a vortex retarder (VR1-442, LBTEK). Polarization with spiral like feature was obtained with other angles of alignment. The laser beam carries different polarization features was focused on the sample in the same condition as described for oriented growth. To obtain arrays of chiral nanostructures, laser beam was scanned across the substrate by moving a piezo stage with step size of 3 μm. After the irradiation, all the samples were rinsed with DI water and blow-dried with nitrogen gun for further characterizations.

### Optical measurements

The scattering spectra of the Au nanospindles were confocally collected through a ×100 DF objective (Olympus, NA = 0.8) with an optical fibre (50 μm) coupled to a spectrometer (QEPro, Ocean Optics).

For CDS measurements, incidence of LCP and RCP light was generated by passing the unpolarized light from the halogen lamp through a linear polarizer and a quarter waveplate with fast axial orientation of ±45° to the linear polarizer (see Supplementary Fig. 20 for detailed setup). The scattering spectra of both LCP and RCP on the chiral nanostructures (*S*_LCP_ and *S*_RCP_) were collected with 50× DF objective (NA = 0.8, Olympus) and the CDS spectra were calculated based on the following definition,

2 $${{{{{\rm{CDS}}}}}}=\frac{2({S}_{{{{{{\rm{LCP}}}}}}}(\lambda)-{S}_{{{{{{\rm{RCP}}}}}}}(\lambda ))}{{S}_{{{{{{\rm{LCP}}}}}}} ({{{{{\rm{\lambda }}}}}})+{S}_{{{{{{\rm{RCP}}}}}}}(\lambda )}$$

Nominal spherical Au NPs were used as a reference for CDS spectra calibration to exclude any artefacts of the circular polarization optics (Supplementary Fig. 21).

For chiral sensing, CDS spectra of chiral Au nanostructures were measured when they were immersed with DI water and the aqueous solution of chiral analytes (5 mg mL^−1^). Between each measurement, the chiral Au nanostructures were immersed in ethanol for 30 min and then rinsed with DI water to detach absorbed chiral molecules.

For the circularly polarized luminescence (CPL) characterizations, the setup is schematically depicted in Supplementary Fig. 22. Specifically, an excitation source of LP CW laser (473 nm) was employed to pump the CdS nanostructures with the power of 20 μW, and the emitted PL were collected confocally to an optofiber spectrometer equipped with a 500 nm long-pass edge filter (FELH0500, Thorlabs) and a circular polarizer (RCP20/LCP20-VIS, Lbtek). For the measurement of *g*_lum_, the PL signals passing through the filters and circular polarizers were sent to a photomultiplier tube (PMT1001/M, Thorlabs) connected to an oscilloscope (DSOX2024A, Keysight). The optoelectric signals were smoothed and processed with following equation to generate *g*_lum_ as

3 $${g}_{{{{{{\rm{lum}}}}}}}=2\frac{{V}_{{{{{{\rm{LCP}}}}}}}-{V}_{{{{{{\rm{RCP}}}}}}}}{{V}_{{{{{{\rm{LCP}}}}}}}+{V}_{{{{{{\rm{RCP}}}}}}}}$$

where *V*_LCP_ and *V*_RCP_ are the PMT output voltage of PL signals detected with LCP and RCP waveplates, respectively.

Unless stated, the CDS and CPL spectra were collected based on single structure of Au and CdS but arrays of 9 individuals for PdO_2−x_, which were fitted with multiple Gaussian peaks. The CDS spectra were recorded over 5–10 chiral nanostructures made at the same irradiation condition to statistically verify the reproducibility of the results. The influence of the objective NA on the CDS spectra is negligible in the measurements (Supplementary Fig. 23).

### Characterizations

SEM images and energy dispersive X-ray Spectroscopy (EDX) of the oriented and chiral nanostructures were captured at the acceleration voltage of 5 and 20 kV respectively. The valance states of the elements in the nanostructures were determined by X-ray photoelectron spectroscopy (XPS, ESCALAB 250Xi, Thermo Fisher Scientific, Waltham, MA, USA). All atomic force microscopy (AFM) measurements of the surface profile were performed with a LensAFM (Nanosurf).

### Simulations

Finite-difference time domain method was applied to simulate the scattering spectra and near field distribution of Au NPs and chiral structures. Specifically, the total field scattering field was applied as the light source, perfectly matched layer was adopted as the boundary condition and the mesh size for Au NPs and chiral structures were 0.5 nm and 3 nm respectively. The refractive index of gold was adopted from Johnson-Christy. The optical forces were calculated based on solving Maxwell tensor and the local temperature profile of the irradiation was calculated based on electromagnetic wave and heat transfer equations using finite element method^57,58^. The thermal conductivity of water and Au are set as ≈0.64 and 318 W m^−1^ K^−1^, respectively. See Supplementary Notes 5, 6 for further details of the simulations.

### Reporting summary

Further information on research design is available in the Nature Portfolio Reporting Summary linked to this article.

## Supplementary information

Supplementary Information

Reporting Summary

## Data Availability

All data generated or analysed during this study are available from the corresponding author upon request. Source data are provided with this paper.

## Source data

Source Data
