# Supplementary material for: Polarization-directed growth of spiral nanostructures by laser direct writing with vector beams
Source: Nat Commun. 2023 Mar 14;14:1422. doi: 10.1038/s41467-023-37048-0 (PMC10015062; doi:10.1038/s41467-023-37048-0)
Supplement: Supplementary file 1 — Supplementary Information [file 41467_2023_37048_MOESM1_ESM.pdf]

Supplementary Information

Polarization-directed growth of spiral nanostructures by laser direct writing with vector beams

Xiaolin Lu, Xujie Wang, Shuangshuang Wang, and Tao Ding*
Key Laboratory of Artificial Micro/Nano Structure of Ministry of Education, School of Physics and Technology, Wuhan University, Wuhan, 430072, China
*Email: t.ding@whu.edu.cn

Table of Content

Supplementary Notes 2

Supplementary Figures 7

Supplementary References 21

Supplementary Notes

Supplementary Note 1. Photochemistry of inorganic materials

The photochemistry of inorganic materials can be generally divided into two categories, direct photolysis and photosensitization[1]. Since we did not use any organic ligands here, the mechanism of the metal nanoparticle (NP) growth is mainly the direct photolysis, which reduces the metal ions with the illumination of laser beam. Typical examples are noble metal ions and complexes such as $[AuCl_4]^-$, Ag^+, etc, which can be photochemically reduced via the reaction pathway as follow[2]

$$\mathrm{AuCl_4^-} + \mathrm{hv} \rightarrow \mathrm{AuCl_3^-} + \mathrm{Cl^{\cdot}} \quad (1)$$

$$\mathrm{2AuCl_3^-} \rightarrow \mathrm{AuCl_3^-} + \mathrm{AuCl_2^-} \quad (2)$$

$$\mathrm{AuCl_2^-} + \mathrm{hv} \rightarrow \mathrm{Au^0} + \mathrm{Cl^-} + \mathrm{Cl^{\cdot}} \quad (3)$$

$$\mathrm{nAu^0} \rightarrow \mathrm{(Au^0)_n} \quad (4)$$

For the metal complexes of $[PdCl_x]^-$ (x=4 or 6), dissociation of Cl^- ion facilitates the photoaquation related to the back electron transfer in the geminate pair via the formula of (5) and (6)[3], which eventually form metal oxides NPs as confirmed by the EDX and XPS characterization (see supplementary Fig. 8).

$$\mathrm{[PdCl_6]^{2-}} + \mathrm{hv} \rightarrow \mathrm{[PdCl_5]^{2-}} \cdots \mathrm{Cl^{\cdot}} + \mathrm{H_2O} \rightarrow \mathrm{[PdCl_5(H_2O)]^{2-}} \cdots \mathrm{Cl^{\cdot}} \quad (5)$$

$$\mathrm{[PdCl_5(H_2O)]^{2-}} \cdots \mathrm{Cl^{\cdot}} \rightarrow \mathrm{[PdCl_5(H_2O)]^-} + \mathrm{Cl^-} \quad (6)$$

For metal chalcogenide such as CdS, their precursors normally first yield the chalcogenide ions via photochemical redox reaction of the chalcogenide

compounds, which then combines with the metal ions to form metal chalcogenide precipitation[4]. For instance, the CdS nanoparticle can be photochemically synthesized via the following pathway[5], which is again confirmed with EDX and XPS (see supplementary Fig. 9).

$$S_2O_3^{2-} + hv = S + SO_3^{2-} \tag{7}$$

$$2S_2O_3^{2-} + hv = S_4O_6^{2-} + 2e^- \tag{8}$$

$$Cd^{2+} + S + 2e^- = CdS \tag{9}$$

The NPs formed initially are all deposited in the center of the beam spot with distribution of Gaussian, which suggests its growth kinetics is proportional to the light intensity (see supplementary Fig. 8-9a). As irradiation proceeds, the individual NPs preferentially merges along the polarization to form nanospindles (see supplementary Fig. 8-9b). These nanospindles further merge laterally to form nanoellipsoids (see supplementary Fig. 8-9c). The mechanism for such oriented growth may also involve some optical dipole interactions as their light field enhancement is an order of magnitude smaller as compared to Au.

Supplementary Note 2. Optical dipole force calculation

The optical induced dipole forces between Au NPs (*r*=7 nm) at the focal plane of the Gaussian beam can be calculated using following formula[6],

$$\mathbf{F} = \int_S \mathbf{T} \cdot \mathbf{n} dS \tag{10}$$

Here **T** is defined as

$$\mathbf{T} = \frac{1}{2} Re\left\{\varepsilon\mathbf{E}\bar{\mathbf{E}} + \mu\mathbf{H}\bar{\mathbf{H}} - \frac{1}{2}(\varepsilon\mathbf{E}\cdot\bar{\mathbf{E}} + \mu\mathbf{H}\cdot\bar{\mathbf{H}})\mathbf{I}\right\} \tag{11}$$

where ε and μ are the permittivity and permeability of the medium, **E** and **H**

are electric and magnetic fields with $\bar{\mathbf{E}}$ and $\bar{\mathbf{H}}$ being their conjugates. **T** is Maxwell's stress tensor, *S* is the surface area of the Au NP, **n** is the unit vector normal to the surface and **I** stands the 3×3 identity matrix. We vary the separation (*r*) between two Au NPs and the optical forces along X, Y and Z directions in space are plotted in Fig. 2h.

The optical dipole potential can be calculated as

$$\mathrm{U_{dp}} = -\int_{\infty}^{\mathrm{r_0}} \mathbf{F}(r)\,\mathrm{d}r \quad (12)$$

Supplementary Note 3. Colloidal force calculations

The Au nanoseeds generated experiences mainly the van der Waals and weak electrostatic interaction which can be described by DLVO theorem. The van der Waals interaction can be described using the following equation[7]

$$U_{\mathrm{vdW}} = -\frac{H_{\mathrm{Au}}}{6}\left(\frac{2r^2}{d(d+4r)} + \frac{2r^2}{(d+2r)^2} + \mathrm{In}\frac{d(d+4r)}{(d+2r)^2}\right) \quad (13)$$

where $H_{\mathrm{Au}}$is the Hamaker constant of Au (≈10×10^{-20} J), *r* is the radius of Au NPs (7 nm), *d* is the separation between two Au NPs.

The electrostatic potential can be calculated with

$$U_{\mathrm{sc}} = 2\pi\varepsilon r\varphi_0^2[1 + \exp(-\frac{d}{L_{\mathrm{d}}})] \quad (14)$$

where ε is the permittivity of the medium, $L_{\mathrm{d}}$is the Debye length, $\varphi_0$is the zeta potential of Au NPs, *d* is the separation between the Au NPs, *r* is the radius of Au NPs.

Thus, the total energy is $\Psi = U_{\mathrm{vdW}} + U_{\mathrm{sc}} + U_{\mathrm{dp}}$, which is plotted as Fig. 2i.

Supplementary Note 4. Vector beam generation

Vector beam is a non-uniformly polarized light, which has different polarization states at each point in the cross section of the beam. Typical vector beam includes radial, azimuthal polarization and hybrid vortex beam. In this work, we generate different polarization configurations with vortex plate which retards the phase of the propagating beam by half a wavelength over the through-aperture.

The Jones matrix for a vortex plate is[8]

$$J(x,y) = \begin{bmatrix} \cos\varphi(x,y) & \sin\varphi(x,y) \\ \sin\varphi(x,y) & -\cos\varphi(x,y) \end{bmatrix} \quad (15)$$

The expression of the polarization of linearly polarized light propagating along the Z-axis at an angle of α with the X-axis

$$E_{int}(x,y) = E_0 \begin{bmatrix} \cos\alpha \\ \sin\alpha \end{bmatrix} \quad (16)$$

After passing through the vortex plate, it can be described using the following equation,

$$E_{out}(x,y) = J(x,y)E_{int}(x,y) = E_0 \begin{bmatrix} \cos\varphi(x,y)\cos\alpha + \sin\varphi(x,y)\sin\alpha \\ \sin\varphi(x,y)\cos\alpha - \cos\varphi(x,y)\sin\alpha \end{bmatrix} \quad (17)$$

where φ is the angle between the fast axis of any point on vortex plate and the x-axis. In our experiment, the distribution of the fast axis on the vortex plate is expressed as $\Phi = \frac{1}{2}\varphi + \beta$, where Φ is fast axis orientation at arbitrary positions of vortex plate, β is the fast axis orientation at $\varphi = 0$. The angle between the polarization and the fast axis of vortex retarder is defined as $\theta = \alpha - \beta$.

Supplementary Note 5. Temperature calculation

Temperature distribution of the Au films during the irradiation is calculated based on the multiphysics coupling of electromagnetic waves and heat transfer in solid with finite element method. The heat equation describes the time-dependent heat transfer as:

$$\rho(\mathbf{r})c(\mathbf{r})\frac{\partial \mathrm{T}(\mathbf{r},t)}{\partial t} = \nabla \cdot [\mathrm{k}(\mathbf{r})\nabla \mathrm{T}(\mathbf{r},t)] + \mathrm{q}(\mathbf{r},t), \tag{18}$$

where $\mathbf{r}$ and t are the coordinate and time respectively, $\mathrm{T}(\mathbf{r},t)$ is the local temperature and the material parameters $\rho(\mathbf{r})$, $\mathrm{c}(\mathbf{r})$, and $\mathrm{k}(\mathbf{r})$ are the mass density, specific heat, and thermal conductivity respectively, $q(\mathbf{r},t)$ means the heat source, which is measured in W m^{-3} and equal to the total power dissipation generated by the electromagnetic field.

Supplementary Note 6. FDTD Simulation Model of Au chiral nanostructure

FDTD method was applied to simulate the scattering spectra of chiral structures. The chiral structure model consists of a spiral arm rotated 10 times around the center. The whole structure is placed on top of the gold film. The geometry of Au chiral nanostructures is shown in the inset of Supplementary Fig. 13.

Supplementary Figures

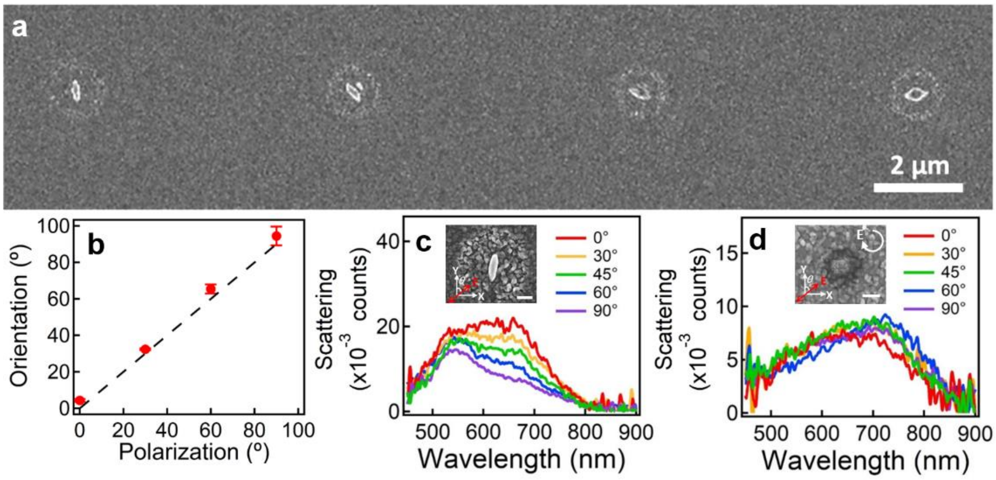

Supplementary Fig. 1 Polarization-dependent optical property of Au spindles. a, SEM image of Au spindle with rotating orientation. **b**, change of the long axis orientation of the Au nanospindle with the laser (446 nm) polarization. Polarization dependent scattering spectra of **c**, Au spindle and **d**, Au NP aggregates made via linear and circular polarized light. Insets are corresponding SEM images. Scale bars are 100 nm. Source data are provided as a Source Data file.

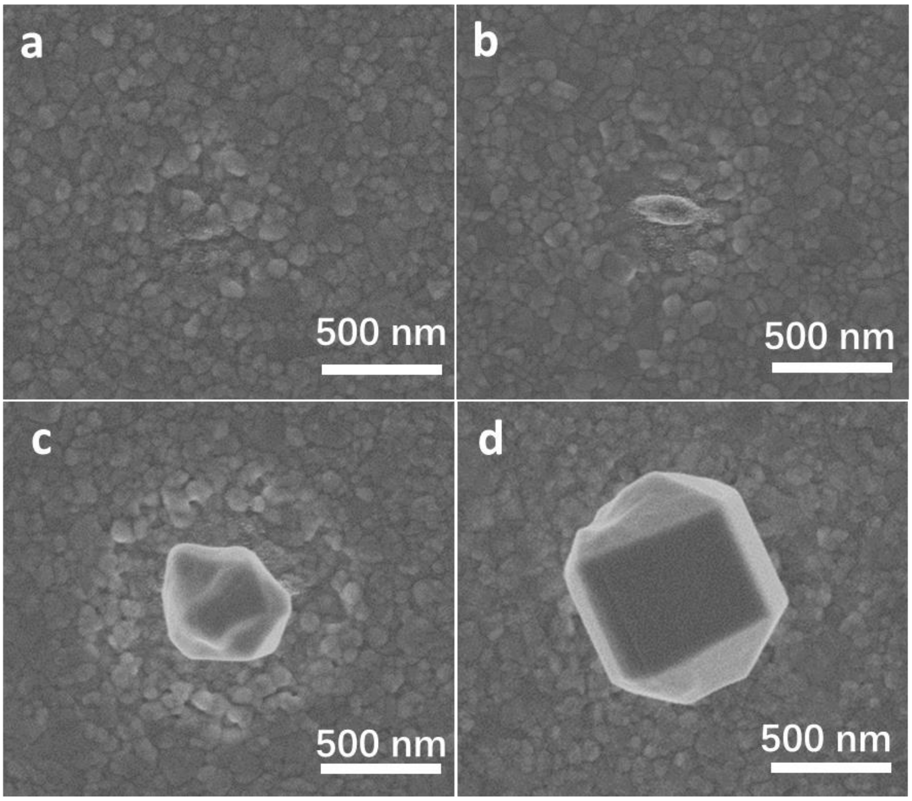

Supplementary Fig. 2 SEM images of Au films immersed in $HAuCl_4$ solution (10 mM) irradiated with different powers. a, 1 mW, **b,** 2 mW, **c,** 3 mW, **d,** 4 mW. The irradiation time is 20 s.

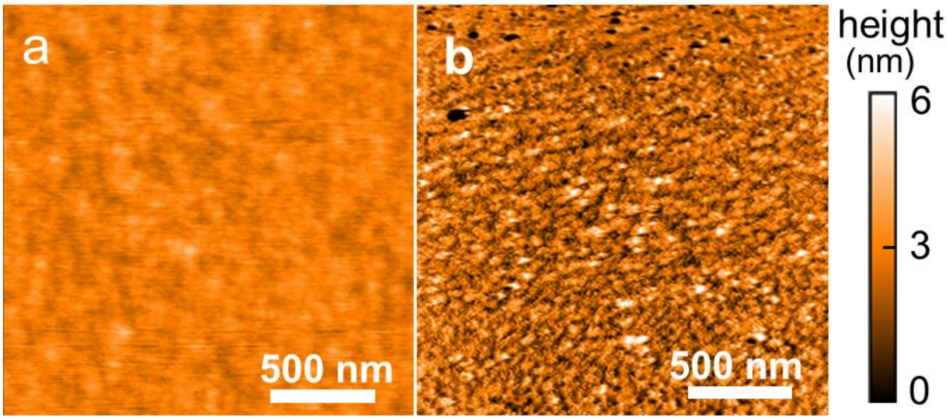

Supplementary Fig. 3 AFM profile of Au films (a) before and (b) after immersion in $HAuCl_4$ solution irradiated with laser (446 nm, 3 mins, ≈10 μW μm^{-2}) for 3 mins. The average surface roughness are **a,** ≈250 pm and **b,** ≈750 pm, respectively.

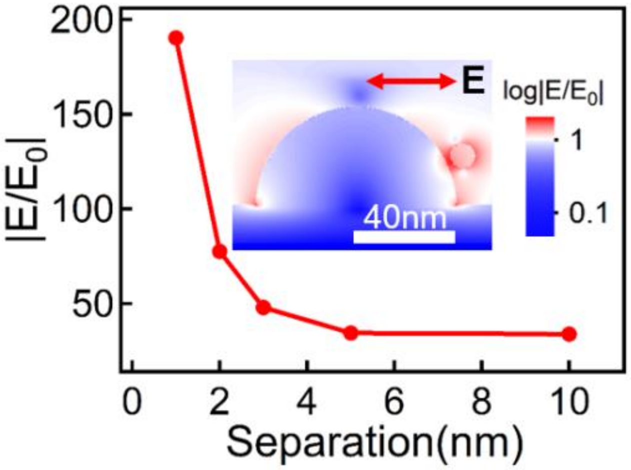

Supplementary Fig. 4 Change of near field intensity with the gap of the Au seed and roughened substrate. Inset is the near field profile with gap of 2 nm. Source data are provided as a Source Data file.

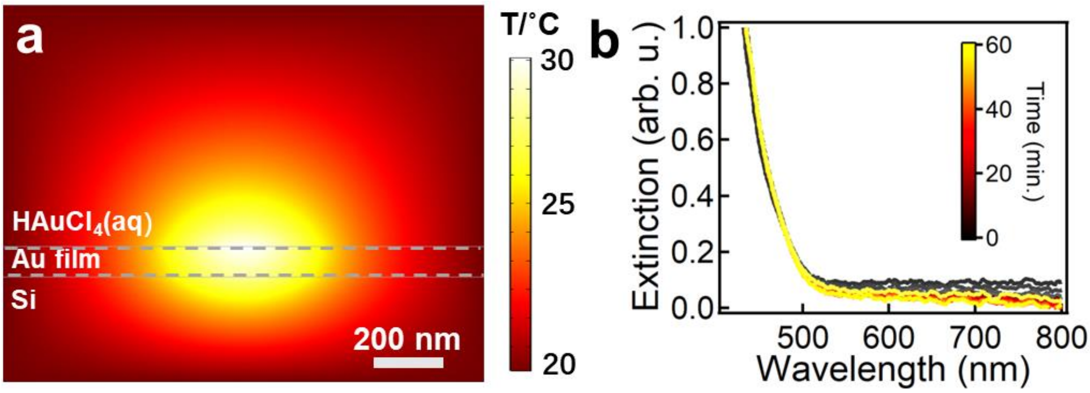

Supplementary Fig. 5 Influence of temperature on the growth of Au NPs. a, temperature distribution of Au film in water medium under 446 nm laser irradiation (4 mW). The bright spot is the focal point of laser beam with waist radius of 270 nm. **b,** kinetic UV-vis spectra of $HAuCl_4$ solution at elevated temperature of 35°C, showing no appearance of plasmon band (≈ 520 nm) during an hour's heating. Source data are provided as a Source Data file.

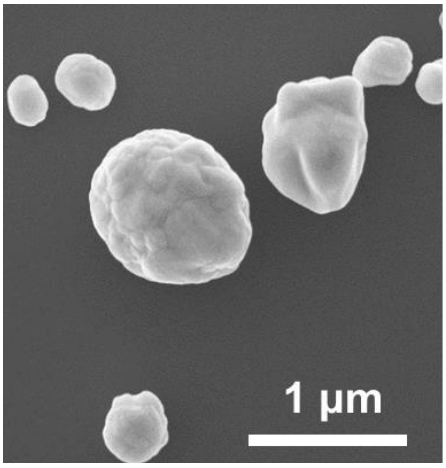

Supplementary Fig. 6 SEM image of Au NPs obtained by irradiating $HAuCl_4$ solution on Si substrate with a linear polarized laser (446 nm).

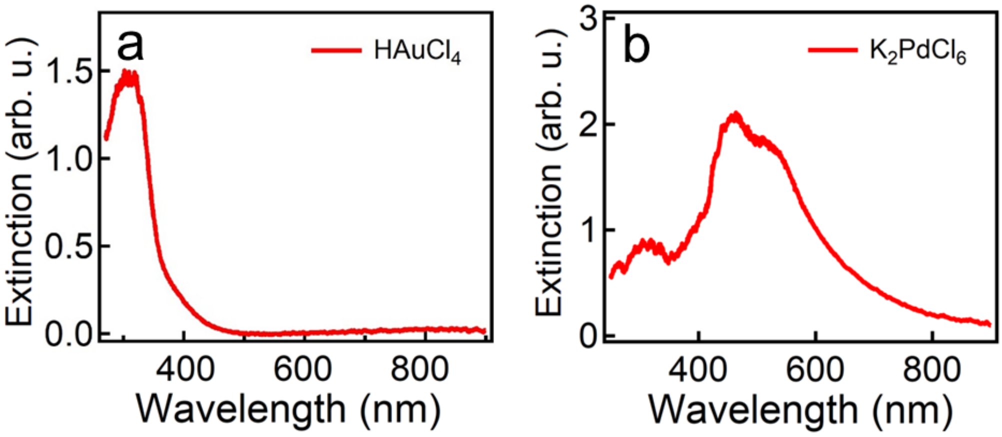

Supplementary Fig. 7 UV-vis extinction spectra of aqueous solution of a, $HAuCl_4$ and **b,** K_2PdCl_6. Source data are provided as a Source Data file.

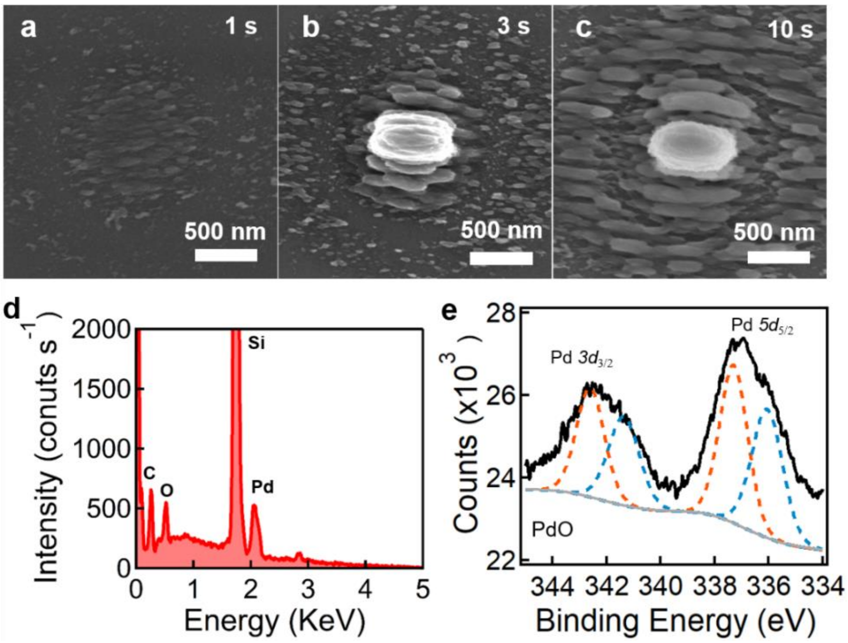

Supplementary Fig. 8 Characterization of $PdO_{2\text{-}x}$ ellipsoids. a-c, Evolution of $PdO_{2\text{-}X}$ nanospindles with irradiation time, laser power: 0.5 mW. **d,** EDX and **e,** XPS spectra of the PdO ellipsoids. Source data are provided as a Source Data file.

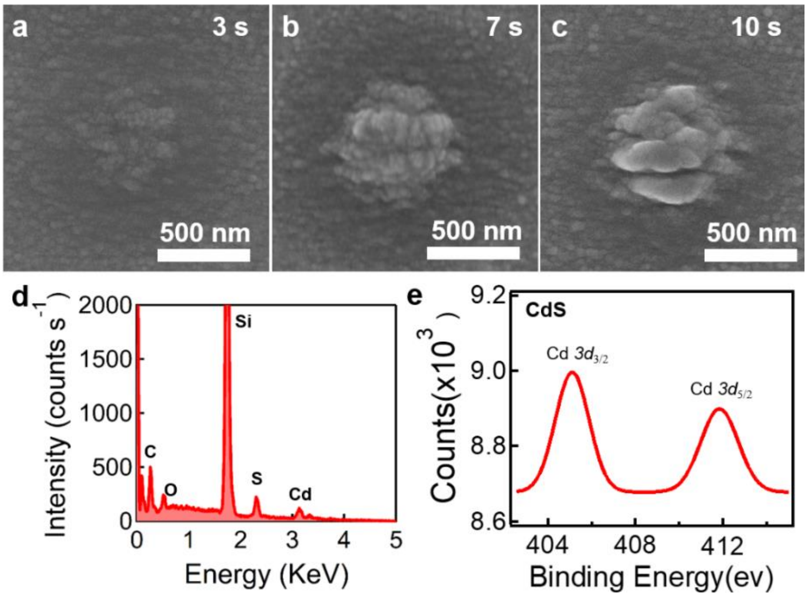

Supplementary Fig. 9 Characterization of CdS ellipsoids. a-c, Evolution of CdS nanospindles with irradiation time, laser power: 2 mW. **d,** EDX and **e,** XPS spectra of the CdS ellipsoids. Source data are provided as a Source Data file.

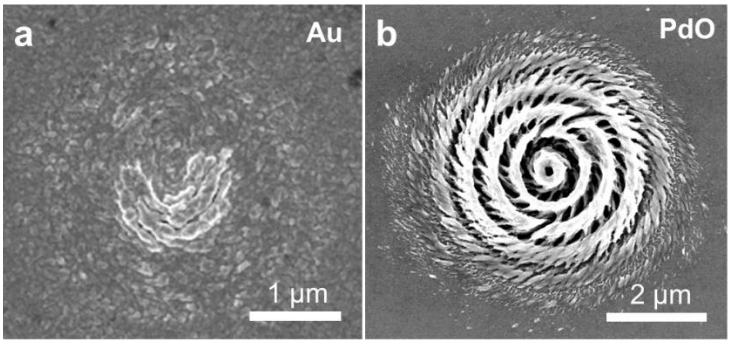

Supplementary Fig. 10 SEM images of other complex nanostructures fabricated with vector beam. **a**, C-shaped Au rings obtained by partially blocking the vector beam; **b**, chiral concentric rings of PdO_{2-x} obtained with long irradiation time.

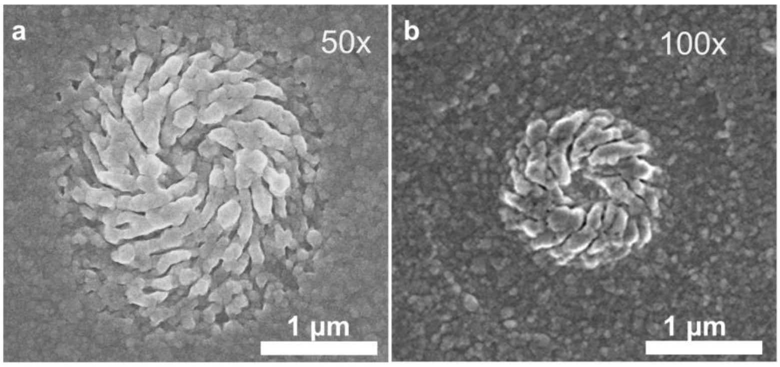

Supplementary Fig. 11 SEM images of Au chiral nanostructures of different diameters obtained with different objective lenses. The irradiation power was 4 mW for both cases. **a**, focusing the beam through 50× objective with irradiation time of 80 s gives a diameter of ≈2 μm; **b**, focusing the beam through 100× objective with irradiation time of 60 s gives a diameter of ≈1.4 μm.

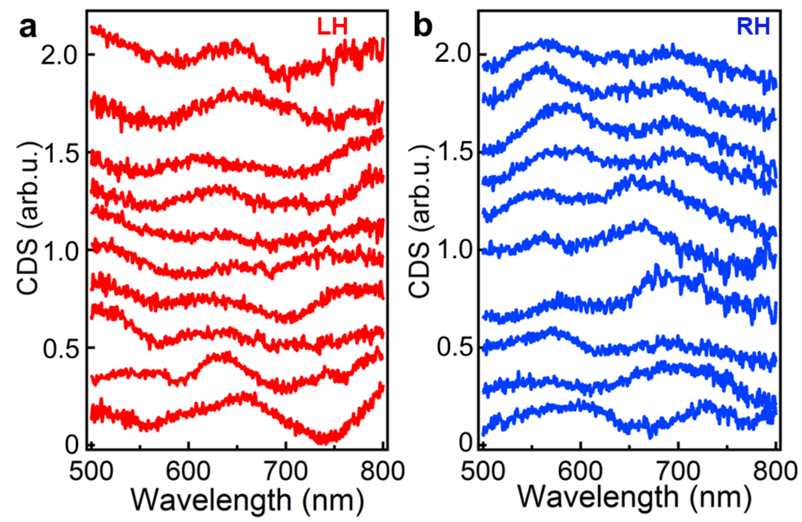

Supplementary Fig. 12 CDS spectra of chiral plasmonic nanostructures made at the same irradiation condition. Source data are provided as a Source Data file.

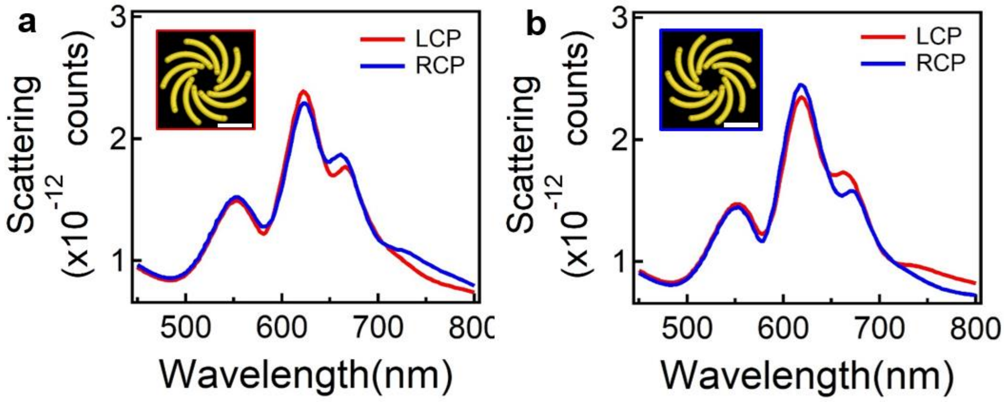

Supplementary Fig. 13 Calculated scattering spectra chiral nanostructures under LCP and RCP normal incidence. a, LH; **b,** RH. Insets are model used for the calculation. Scale bars are 500 nm. Source data are provided as a Source Data file.

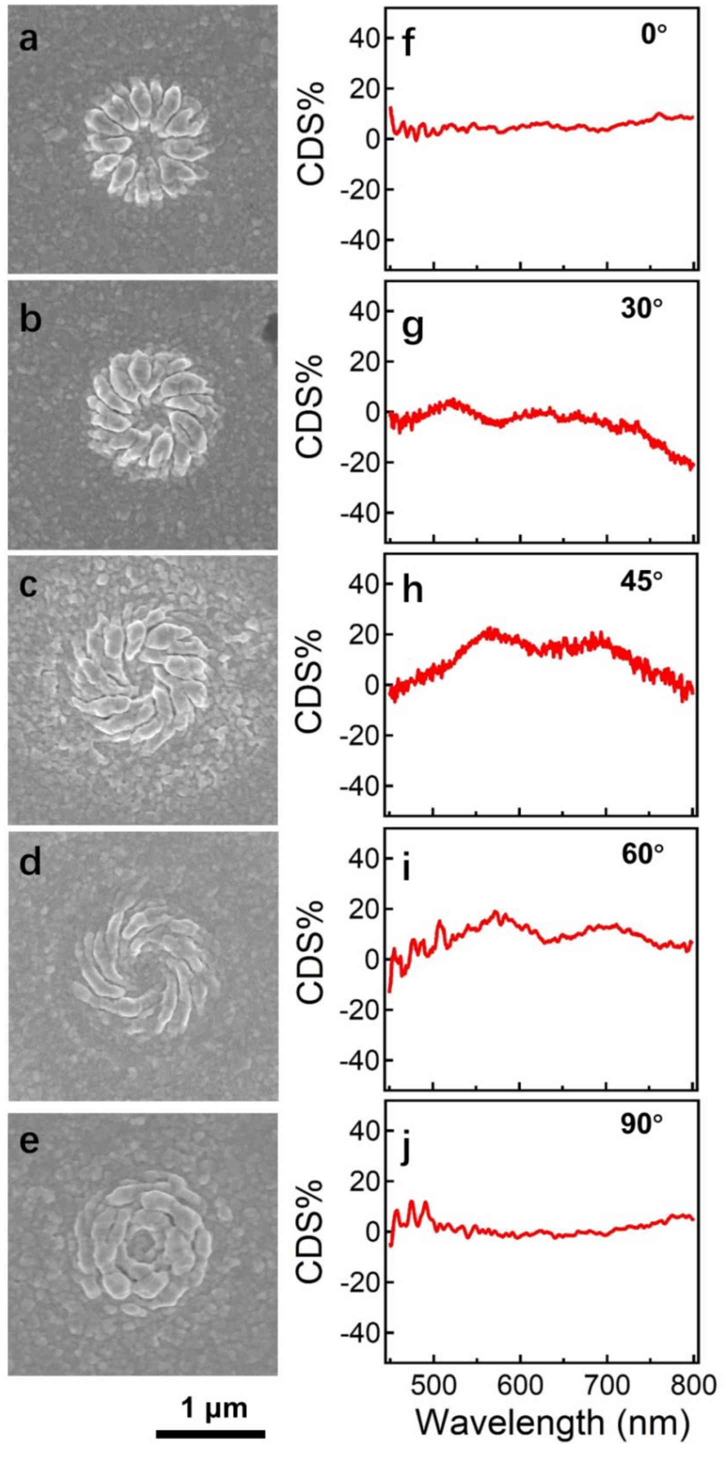

Supplementary Fig. 14 Tuneable chiroptic property of complex Au nanostructures obtained by changing the alignment angle (θ) from 0° to 90°. a-e typical SEM images of the chiral Au nanostructures with θ of **a,** 0°, **b**, 30°, **c**, 45°, **d**, 60°, **e**, 90°, and **f-j**, their averaged CDS spectra over five individual structures. Source data are provided as a Source Data file.

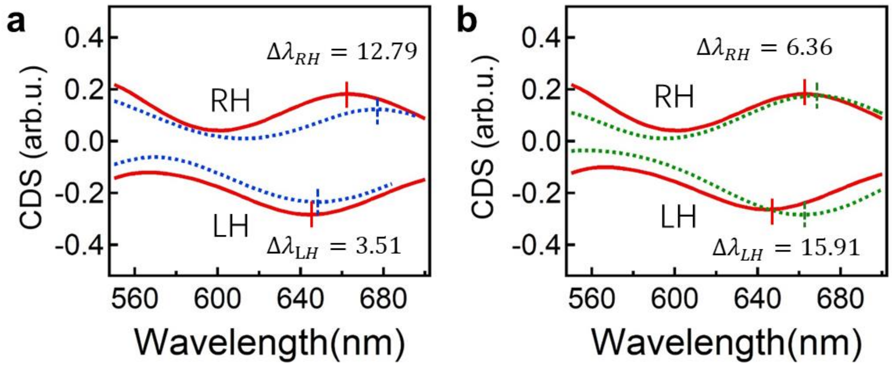

Supplementary Fig. 15 Sensing of L- and D-Cysteine with LH and RH enantiomers of spiral Au nanostructures. a, L-Cysteine; **b,** D-Cysteine. The spectral curves were smoothed (binominal) and fitted (Gaussian multiple peak fit) from the raw data for easy identification of the peak shift. Solid and dash lines are the chiral structure without and with cysteine respectively. Source data are provided as a Source Data file.

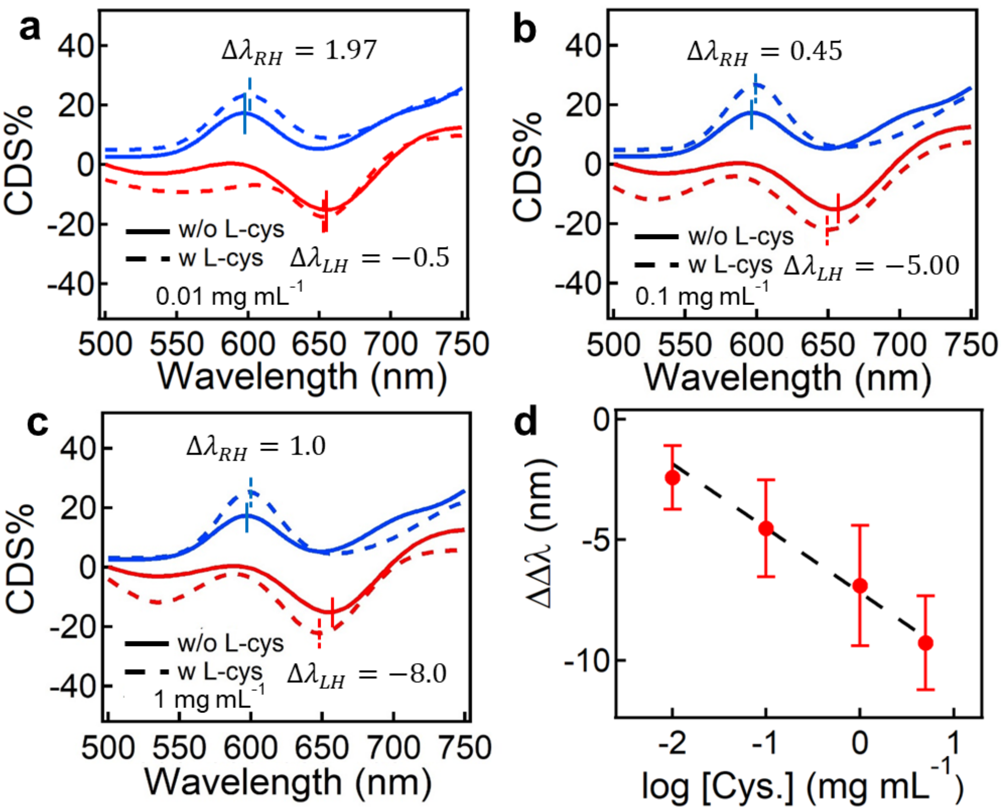

Supplementary Fig. 16 Sensitivity of L-cysteine molecules by the chiral plasmonic nanostructures. CDS spectra of spiral Au nanostructure immersed in with and without L-cysteine of different concentrations **a**, 0.01 mg mL^{-1}, **b**, 0.1 mg mL^{-1}, **c**, 1 mg mL^{-1}. d, change of dissymmetric factor with concentration of L-cysteine. Source data are provided as a Source Data file.

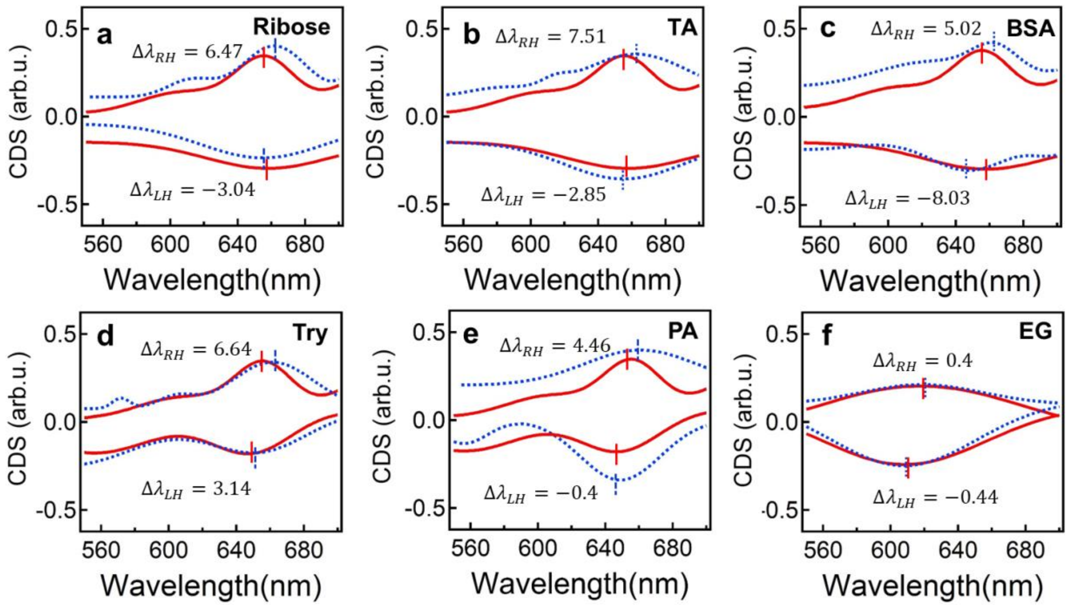

Supplementary Fig. 17 Sensing of chiral molecules with LH and RH enantiomers of spiral Au nanostructures. CDS spectra of LH and RH structures in water (solid lines) with **a,** L-ribose, **b,** L-Tractic acid (TA), **c,** L- BSA, **d,** L-Trypotophan (Try), **e,** L-Phenylalanine (PA), **f,** 1,2-Ethanediol (also known as ethylene glycol, EG) (dash lines). Note different sets of spiral Au nanostructures might be applied for the measurements. Source data are provided as a Source Data file.

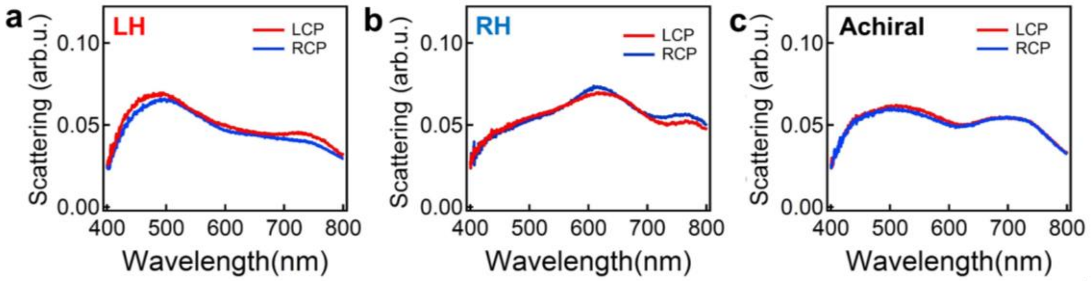

Supplementary Fig. 18 Scattering spectra of PdO_{2-x} nanopillar arrays with LCP and RCP incidences. a, LH, **b,** RH and **c,** achiral structure under LCP and RCP light. Source data are provided as a Source Data file.

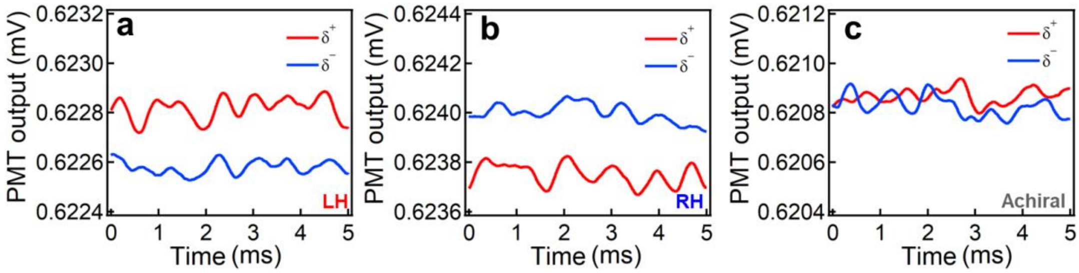

Supplementary Fig. 19 Optoelectronic detection of chiral emission from LH, RH and achiral CdS nanostructures with excitation of linearly polarized (LP) light (473 nm). The PL signals from the CdS samples first pass through a left or right circular polarizer (denoted as δ^+ and δ^-), and then a 600 nm long pass and 700 nm short pass filters before reaching the PMT (Supplementary Fig. 22). The asymmetric output in the achiral samples was normalized to zero to reflect the real g_{lum} of the chiral samples. Source data are provided as a Source Data file.

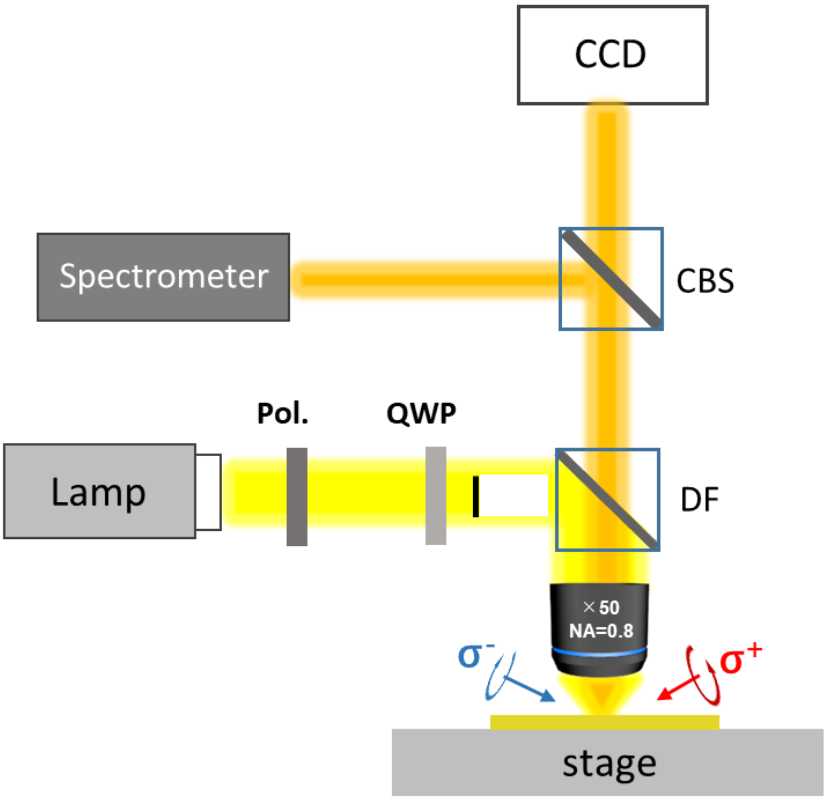

Supplementary Fig. 20 Scheme of CDS measurement setup. Pol.: polarizer, QWP: quarter waveplate, CBS: cube beam splitter, DF: dark field.

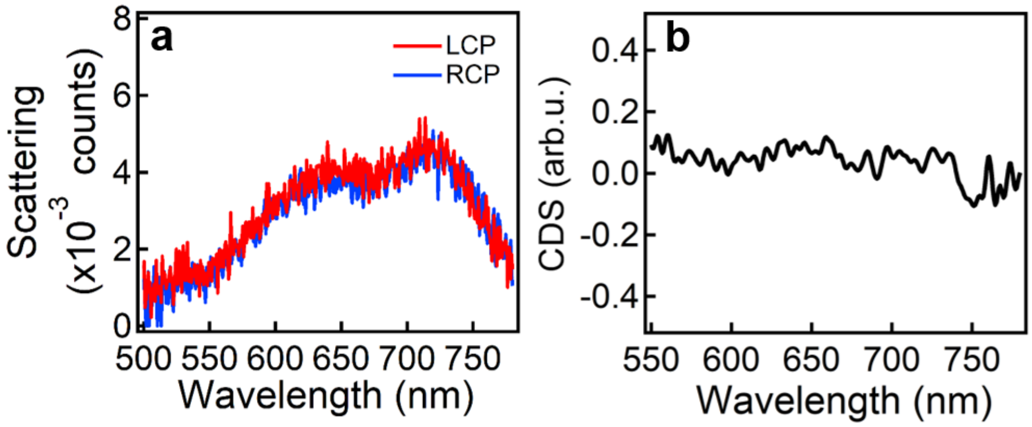

Supplementary Fig. 21 Nominal spherical Au NP as a reference. a, Scattering spectra of spherical Au NPs (80 nm) with LCP and RCP incidences and **b,** the corresponding CDS spectrum. Source data are provided as a Source Data file.

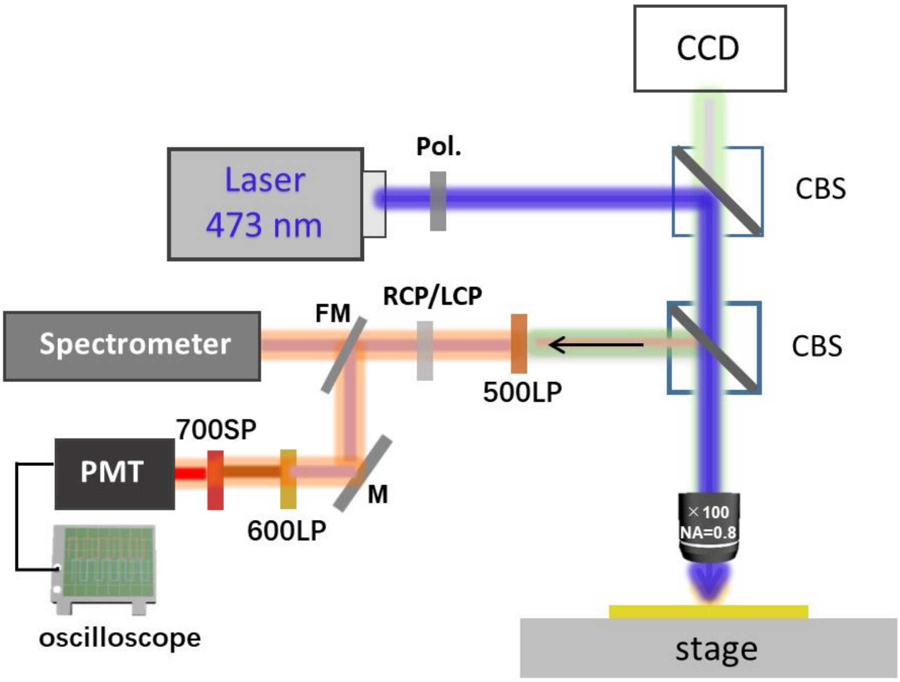

Supplementary Fig. 22 Scheme of the chiral emission measurement setup. Pol.: polarizer, FM: Flip mirror, M: mirror, CBS: cube beam splitter, LP/SP: long/short pass filter. RCP/LCP: right/left circular polarizers mounted on motorized switch.

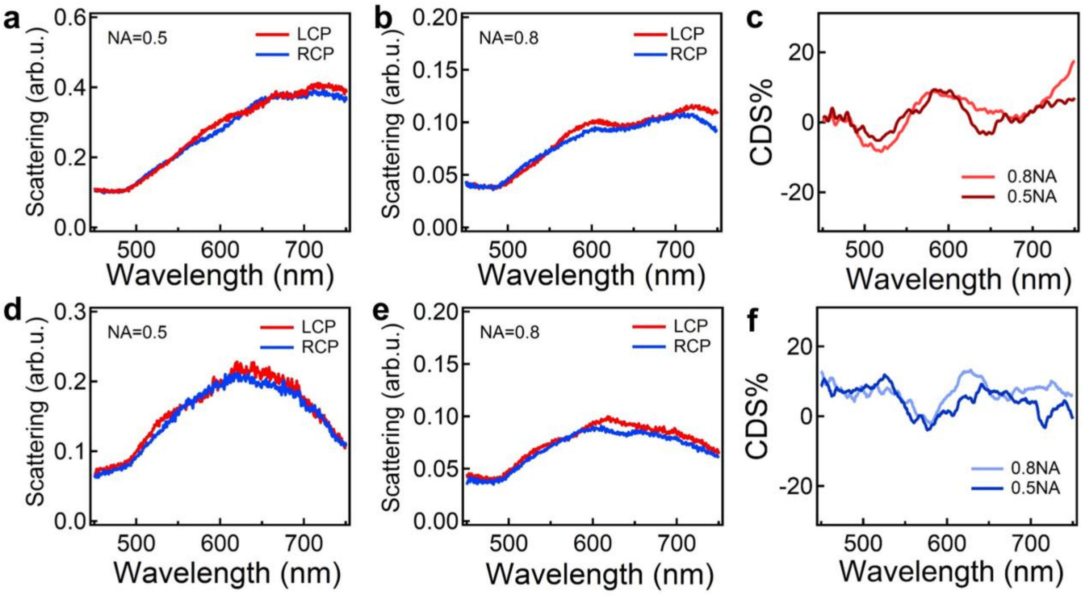

Supplementary Fig. 23 Comparison of CDS spectra measured from NA=0.5 and NA=0.8. a-c, LH and d-f, RH structures. **a,b,d,e** are dark field scattering spectra and **c, f** are CDS spectra. Source data are provided as a Source Data file.

Supplementary References

1. Sakamoto M., Fujistuka M., Majima T. Light as a construction tool of metal nanoparticles: Synthesis and mechanism. *J. Photochem. Photobiol. C* **10**, 33-56 (2009).

2. Yang S., Wang Y., Wang Q., Zhang R., Ding B. UV irradiation induced formation of Au nanoparticles at room temperature: The case of pH values. *Colloids Surf. A Physicochem. Eng. Asp.* **301**, 174-183 (2007).

3. Znakovskaya I. V., Sosedova Y. A., Glebov E. M., Grivin V. P., Plyusnin V. F. Intermediates formed by laser flash photolysis of $[PtCl_6]^{2-}$ in aqueous solutions. *Photochem. Photobiol. Sci* **4**, 897-902 (2005).

4. Lokhande C. D. Chemical deposition of metal chalcogenide thin films. *Mater. Chem. Phys.* **27**, 1-43 (1991).

5. Ichimura M., Goto F., Arai E. Photochemical deposition of Cds from aqueous solutions. *J. Electrochem. Soc.* **146**, 1028-1034 (1999).

6. Li Z., Käll M., Xu H. Optical forces on interacting plasmonic nanoparticles in a focused gaussian beam. *Phys. Rev. B* **77**, 085412 (2008).

7. Findenegg G. H. J. N. Israelachvili: Intermolecular and surface forces (with applications to colloidal and biological systems). Academic press, london, orlando, san diego, new york, toronto, montreal, sydney, tokyo 1985. 296 seiten. *Berichte der Bunsengesellschaft für physikalische Chemie* **90**, 1241-1242 (1986).

8. McEldowney S. C., Shemo D. M., Chipman R. A., Smith P. K. Creating vortex retarders using photoaligned liquid crystal polymers. *Opt. Lett.* **33**, 134-136 (2008).
